# Comparative Evaluation of the Clinical Efficacy of PRP-Therapy, Minoxidil, and Their Combination with Immunohistochemical Study of the Dynamics of Cell Proliferation in the Treatment of Men with Androgenetic Alopecia

**DOI:** 10.3390/ijms21186516

**Published:** 2020-09-06

**Authors:** Elena E. Pakhomova, Irina O. Smirnova

**Affiliations:** 1 Department of Infectious Diseases, Epidemiology and Dermatovenereology, St. Petersburg State University, 199034 St. Petersburg, Russia; driosmirnova@yandex.ru; 2 Thichology Center of Hair Treatment, 191123 St. Petersburg, Russia

**Keywords:** platelet-rich plasma (PRP), androgenetic alopecia (AGA), minoxidil, hair morphometry, hair follicle cell proliferation, β-catenin, CD34, Ki67, Dkk-1

## Abstract

Platelet-rich plasma (PRP) therapy has been considered as a promising treatment for androgenetic alopecia (AGA). The aim of the study was comparative evaluation of the clinical efficacy of PRP-therapy, minoxidil, and their combination in the treatment of men with AGA and to evaluate the effects of PRP on the proliferation of hair follicle (HF) cells in skin biopsy. Materials and Methods: The study involved 69 men who were divided into 3 groups who received PRP therapy, minoxidil, and their combination. The clinical efficacy of the therapy was evaluated by the dynamics of morphometric of hairs. To assess cell proliferation antibodies to β-catenin, CD34, Ki67, and to Dkk-1 were used. Results. PRP treatment was more effective than minoxidil therapy (*p* = 0.005). Complex therapy turned out to be more effective than minoxidil monotherapy (*p* < 0.0001) and PRP monotherapy (*p* = 0.007). After applying PRP the absolute and relative values of the β-catenin and CD34 expression area increased; an increase in Ki67+ index was also significant. Conclusions: PRP can be considered as a treatment option for AGA. Combined PRP and minoxidil use seems promising for the treatment of AGA. PRP increase in the proliferative activity of HF cells and improves hair morphology in patients with AGA.

## 1. Introduction

Currently one of the most discussed treatments of androgenetic alopecia (AGA) is platelet-rich plasma (PRP) therapy. There are few studies, including randomized, placebo-controlled studies, in which the positive effects of PRP injections on hair growth and their morphometric parameters in AGA have been obtained [1,2,3,4,5,6]. The main clinical results obtained in the course of studies were manifested in the decrease in hair loss and an increase in hair density. However, to assess the clinical effects of PRP therapy, subjective assessment methods are often used-pulling test, questionnaires, survey photographs. Objective methods of assessment-morphometry using trichoscopy and phototrichogram-were used less frequently.

Studies demonstrating the comparative efficacy of PRP in the complex treatment of AGA, for example, in combination with traditional therapy methods (minoxidil, finasteride), are rare [7]. No work has been published on the comparative assessment of the effectiveness of minoxidil monotherapy, PRP monotherapy, and their combination in men with AGA.

In vitro studies demonstrate changes in the presence of PRP in the expression of β-catenin, a key molecule of the Wnt/β-catenin signaling pathway that regulates hair follicle (HF) growth and hair matrix cell proliferation [8,9,10]. Single studies on scalp biopsy samples using markers CD31 and Ki67 showed that PRP improves microcirculation around hair follicles and increases the proliferative activity of HF cells [2,11]. In turn, CD34+ cells in human HF are located below the bulge zone, suprabulbar, as well as in the skin between hair follicles, in the basal cells of the interfollicular epidermis and are manifested during the anagen phase [12]. Studies on the assessment of vascular endothelial proliferation using antibodies to CD34 after PRP application on scalp samples have not been published. Studies assessing the proliferation of HF cells using various markers after PRP application on scalp samples are few.

Despite the positive practical experience of application, studies assessing the clinical effects and mechanisms of PRP action in patients with AGA are still insufficient for a good evidence base for this treatment method [13].

The aim of the study was comparative evaluation of the clinical efficacy of PRP-therapy, minoxidil and their combination in the treatment of men with androgenetic alopecia and to evaluate the effects of PRP on the proliferation of hair follicle cells in skin biopsy.

## 2. Results

### 2.1. Dynamics of Phototrichological Indicators of Hair Growth in Patients of Observation Groups after Treatment

**Figure 1:**
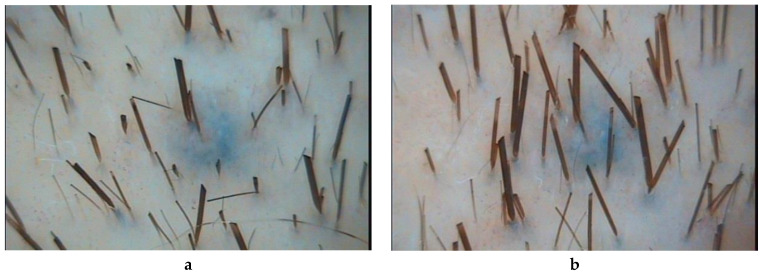
Trichoscopy (phototrichography) of a patient who received an injections of platelet-rich plasma: **a**—before treatment; **b**—after treatment. Magnification ×60.

In patients of observation group 1, who received PRP injections, all parameters of hair growth underwent significant changes. The hair density increased by 12% (*p* = 0.000067), the average hair diameter by 12% (*p* = 0.001947), the proportion of vellus hairs decreased by 17% (*p* = 0.002225), and the proportion of telogen hairs by 16% (*p* = 0.02836) (Table 1, Figure 1).

**Figure 2:**
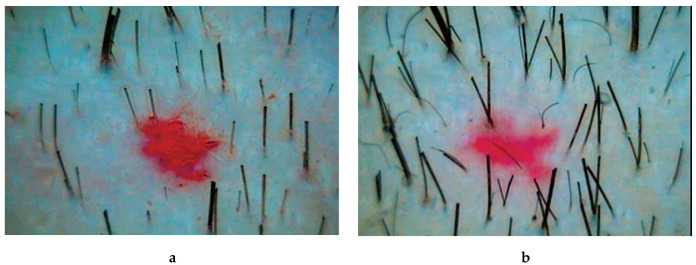
Trichoscopy (phototrichography) of a patient who received complex therapy-applications of a 5% minoxidil solution and PRP injections: **a**—before treatment; **b**—after treatment. Magnification ×60.

In patients of observation group 2, who received complex therapy–application of 5% minoxidil solution and PRP injection–all indicators of hair growth also underwent significant changes. Hair density increased by 32% (*p* = 0.00004), mean hair shaft diameter increased by 26% (*p* = 0.00004). The intensity of hair loss decreased by 39% (*p* = 0.00008), which was combined with a decrease in the proportion of vellus hair by 30% (*p* = 0.00082) (Table 1, Figure 2). 

**Figure 3:**
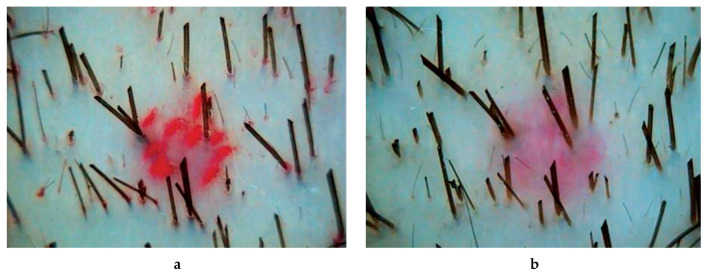
Trichoscopy (phototrichography) of a patient who received standard therapy with a 5% minoxidil solution: **a**—before treatment; **b**—after treatment. Magnification ×60.

In patients of group 3, receiving standard therapy with 5% minoxidil solution, the index of hair density significantly increased—by 16% (*p* = 0.00073) (Table 1, Figure 3).

### 2.2. Comparative Assessment of the Dynamics of Phototrichological Indicators of Hair Growth in Patients of Observation Groups

**Figure 4:**
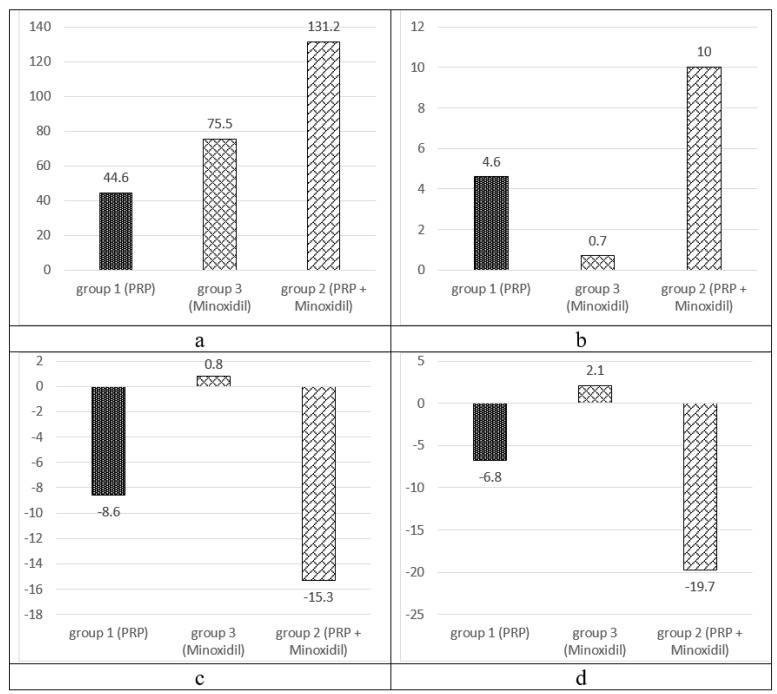
Dynamics of morphometric indicators of hair growth: hair density on 1 cm^2^ (**a**), average diameter of all hair, μm (**b**), share of telogen hair, % (**c**), share of vellus hair, % (**d**).

A comparative assessment of the dynamics of phototrichological parameters in patients of the observation groups is presented in (Figure 4).

In a comparative assessment of the results of treatment in groups 1 and 3, significant differences were not revealed only in terms of hair density, ∆12 and 16%, respectively (*p* = 0.1968) (Table 2). The effectiveness of PRP in comparison with minoxidil in terms of average hair diameter was 6.3 times higher, ∆12 and 2%, respectively (*p* = 0.0248), in terms of the share of vellus-11.8 times, ∆−17 and 2% respectively (*p* = 0.0069), in terms of telogen share-3.1 times, ∆−16 and 5%, respectively (*p* = 0.0311).

The effects of complex therapy exceeded the effects of PRP monotherapy in terms of hair density by 2.9 times (Δ32% and 12%, respectively, *p* = 0.0001), diameter of hair shafts by 2.2 times (Δ26% and 12%, *p* = 0.0071), the proportion of telogen hair by 2.9 times (Δ−39% and −16%, *p* = 0.0025). The difference in the proportion of vellus in the observation group 2, although it exceeded 1.8 times (Δ−30% in group 1 and −17% in group 2), but was not statistically significant, *p* = 0.0715 (Table 3).

In the course of a comparative analysis, it was found that the features of complex therapy in comparison with traditional therapy with minoxidil were an increase in hair density by 1.74 times (Δ32% and 16%, respectively, *p* = 0.0347), an increase in the thickness of hair shafts by 14.3 times. (Δ26% and 2%, respectively, *p* = 0.00001). Decrease in the proportion of telogen hair by 9.3 times (Δ−39% and +5%, respectively, *p* = 0.00003), decrease in the proportion of vellus hair by 19.1 times (Δ−30% and +2%, respectively, *p* = 0.0009) (Table 4).

### 2.3. Results of a Comparative Assessment of the Clinical Efficacy of Therapy Using PRP

The quartile distribution of the dynamics of hair growth indicators for patients of all groups after treatment is presented in Table 5.

In the group of patients receiving PRP therapy, 9 patients (36% each) showed no dynamics for each of the hair growth parameters. An improvement in terms of hair density was recorded in 9 people (36%), according to the average hair diameter—in 9 people (36%), according to the share of telogen hair—in 9 people (36%), and according to the share of vellus hair—in 10 people (40%). A significant improvement in terms of density, average diameter, and proportion of telogen hair was observed in 7 people (28% each), according to the proportion of vellus hair—in 6 patients (24%).

In the group of patients receiving PRP and minoxidil in complex therapy, no dynamics in terms of hair density was observed in 1 patient (4.5%), in the proportion of vellus hair—in 2 patients (9%), in terms of the average hair diameter and the proportion of telogen hair such patients were absent. An improvement in terms of hair density was observed in 13 patients (59%), in terms of the average hair diameter—in 11 patients (50%), in the proportion of telogen hair—in 13 patients (59%), and in the proportion of vellus hair—in 10 patients (45%). Significant improvement in terms of hair density was observed in 8 patients (36%), in terms of average hair diameter—in 11 patients (50%), in the proportion of telogen hair—in 9 patients (41%), and in the proportion of vellus hair—in 10 patients (45%).

In the group of patients receiving minoxidil, no dynamics in terms of density and average hair diameter was observed in 7 patients (32% each), in the proportion of telogen hair—in 8 patients (36%), and in the proportion of vellus hair—in 6 patients (27%). An improvement in hair density was observed in 11 patients (50%), in the average hair diameter in 15 patients (68%), in the proportion of telogen hair in 12 patients (54%), in the proportion of vellus hair in 14 patients (63%). A significant improvement in terms of hair density was observed in 4 people (18%), according to the share of vellus and telogen hair—2 people each (9% each), no significant improvement in the average hair diameter was observed in any patient.

When evaluating clinical efficacy in group 3 of patients treated with minoxidil, improvement after treatment was observed in 13 patients (59%), and no dynamics was observed in 9 patients (41%).

In the first group of patients receiving PRP-therapy, the treatment was accompanied by an improvement in 7 patients, which amounted to 28% (of which degree I AGA was observed in 1 patient, degree III—in 4 patients, degree IV-in 2 patients); significant improvement—in 9 patients (36%, of which degree I AGA was observed in 1 patient, degree II—in 2 patients, degree III—in 5 patients, degree IV—in 1 patient); and no dynamics—in 9 patients (36%, 4 patients had degree III, 5 patients had degree IV AGA), (Table 6). Thus, PRP treatment was more effective than minoxidil therapy (χ^2^ = 10.652, *p* = 0.005).

In the group of patients receiving combined therapy with PRP and minoxidil (group 2), treatment was accompanied by an improvement in 8 patients, which was 36% (all patients had degree IV AGA); significant improvement—in 14 patients, which amounted to 64% (of which degree I was observed in 1 patient, 4 patients with degrees II, III, and IV, and degree V was observed in 1 patient). Thus, complex therapy turned out to be more effective than minoxidil monotherapy (χ^2^ = 24.190, *p* < 0.0001) and PRP monotherapy (χ^2^ = 10.003, *p* = 0.007). The lack of dynamics was not observed in any patient.

### 2.4. Evaluation of Markers of Proliferative Activity of HF Cells after PRP Therapy

**Figure 5:**
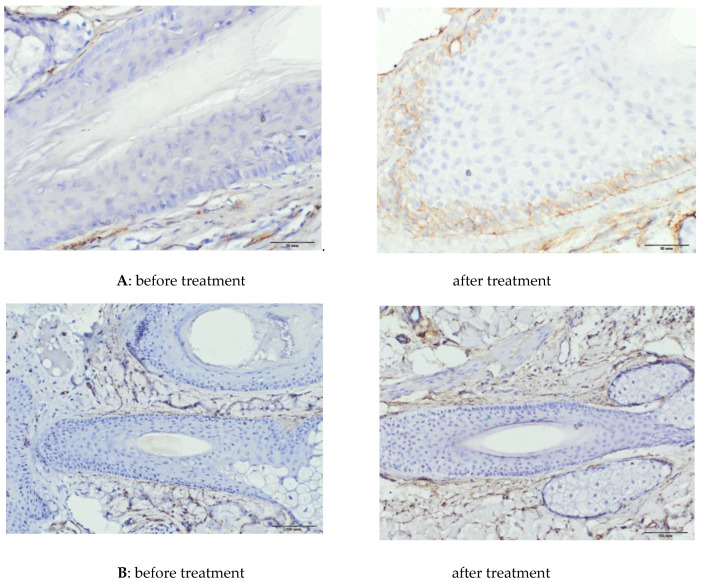
Area of CD 34. (**A**): Area of CD 34+ in the outer root sheaths of the HF, magnification ×400. (**B**) Area of CD 34+ in the interfollicular matrix, magnification ×200.

**Figure 6:**
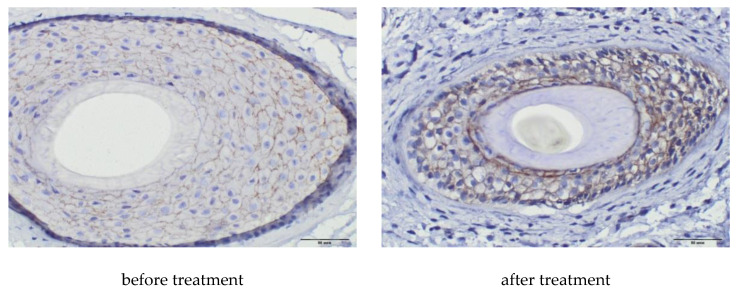
Area of β-catenin, magnification ×400.

**Figure 7:**
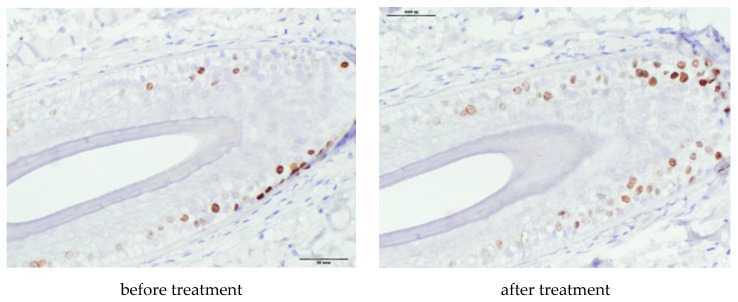
Number of Ki 67+ cells, magnification ×400. Dkk-1 expression was absent in all medicines before and after treatment.

In an immunohistochemical study after treatment, the absolute and relative values of the area of expression of CD34 (∆287%, *p* = 0.0001 and 325%, *p* = 0.0003, respectively) underwent significant positive changes (Table 7, Figure 5), as well as absolute and relative values of the area of β-catenin expression (∆165%, *p* = 0.0306 and ∆96%, *p* = 0.0018, respectively), (Table 7, Figure 6). The increase in the Ki67+ index was also significant (∆191%, *p* = 0.0111), with a tendency to an increase in the number of immunopositive nuclei in HF cells (Table 7, Figure 7).

## 3. Discussion

PRP therapy is a simple, cost-effective and feasible treatment for hair loss and can be considered as an alternative treatment for AGA. In the course of our study, it was demonstrated that in patients receiving PRP injections, all indicators of hair growth underwent significant changes: an increase in hair density and their average diameter by 12%; the share of vellus hair decreased by 17%, and the share of telogen hair by 16%, which is consistent with the data of studies of the effectiveness of PRP published in the world literature. In our study, 47 patients received PRP-therapy, only 4 of them (8.5%) complained of pain at the injection sites, which was not a reason for canceling the procedures in any case. No other side effects were reported.

In patients of the control group, receiving standard therapy with 5% minoxidil solution, the index of hair density significantly increased (by 16%). In a comparative assessment of the treatment results in the PRP and minoxidil groups, significant differences were not revealed only in terms of hair density, ∆12 and 16%, respectively. For all other indicators of hair growth, the efficiency of PRP was significantly higher: in terms of the average hair diameter, it was 6.3 times higher; in terms of the share of vellus-11.8 times; by the share of telogen-3.1 times. The superior effect of PRP over minoxidil may have been due to the fact that the average follow-up is short, and further studies with longer follow-up are needed to draw fundamental conclusions, including the long-term efficacy of treatment. Four months is the minimum period for evaluating the effectiveness of minoxidil, it is better to evaluate it in a longer period (6–12 months). However, our data indicate that PRP also has a delayed effect and in some cases, with a longer follow-up period (8–12 months), shows an improvement in the dynamics of hair growth. Navarro and colleagues retrospectively compared growth factor-rich plasma (PRGF) versus topical minoxidil therapy for the treatment of AGA, where telogen reduction was greater in the PRGF group [14]. In a very recently published study VermaK. and co-authors also noted that PRP therapy was superior to minoxidil and could be a valuable alternative to topical minoxidil therapy for AGA treatment [15].

In our work, the result of the combined use of PRP and minoxidil was of particular interest. A similar study was published in 2018. AlvesR. and GrimaltR. showed that the use of PRP in combination with the standard treatment method—minoxidil or finasteride-demonstrates a more pronounced clinical effect, confirmed by trichoscopy, compared with the latter monotherapy [7]. Another study by Shah K.B. et al., aimed at comparing the effectiveness of topical minoxidil (5%) and the combined use of minoxidil and dermoroller with PRP injections in men with AGA [16]. The group of patients treated with the combination of minoxidil, PRP and dermoroller showed greater satisfaction with the treatment than the group of minoxidil. The results were assessed 6 months later on the basis of a 7-point questionnaire and survey photographs; the analysis of morphometric data (trichoscopy, phototrichogram) was not carried out.

In the course of our study, we have demonstrated the high clinical efficacy of complex therapy and its advantage over PRP monotherapy and the standard method using minoxidil. In the course of a comparative analysis, it was found that the effectiveness of complex therapy exceeds the effectiveness of traditional therapy with minoxidil in terms of hair density by 1.74 times; the thickness of the hair shafts by 14.3 times; the share of telogen hair is almost 9.3 times, the share of vellus hair is 19.1 times. The effects of complex therapy in our study exceeded the effects of PRP monotherapy in terms of hair density by 2.9 times; diameter of hair shafts 2.2 times; share of telogen hair 2.9 times. Although the difference in the indicator of the proportion of vellus exceeded 1.8 times, was not statistically significant, *p* = 0.0715.

Minoxidil does not alter testosterone levels or adrenal-androgenic secretion, nor does it alter the genetically programmed sensitivity of hair follicles to androgens. PRP and minoxidil prolong the anagen phase, promote proliferation and lifespan of dermal papilla cells during the hair growth cycle by inhibiting apoptosis [8,9,17,18], and seem to potentiate each other’s action when used together. Data on the specific effect on the pathogenesis of AGA have not been obtained, the mechanism of action of minoxidil, as well as PRP, is not fully understood. In 2104, the effectiveness of PRP was shown in the treatment of 11 patients with AGA who were resistant to minoxidil and finasteride therapy [4]. The active metabolite of minoxidil, catalyzed by the enzymes sulfotransferases, minoxidil sulfate, is responsible for stimulating hair growth; resistance to minoxidil is associated, among other things, with low sulfotransferase activity in the scalp. At the same time, another study has shown that platelets exhibit minoxidil sulfotransferase activity [19]. A possible relationship between platelet sulfotransferase activity and high efficiency of PRP in combination with minoxidil is one of the vectors for further study.

Published studies on the effectiveness of PRP have used different preparation protocols with beneficial effects. Due to the lack of a single standard protocol for the preparation of PRP and the method of AGA treatment at the moment, we prepared PRP by the method of double centrifugation with “soft spin”, in which layers of blood cells were manually separated, and we also applied platelet activation through coagulation, which causes the secretion of various growth factors and potentiates mitogenic effects in different cell types. Haynesworth et al. [20] showed that proliferation and differentiation of mesenchymal stem cells in adults correlated with platelet concentration: a sufficient cellular response began with an increase of 4–5 times the initial number. A similar study by Y. Lui and co-authors [21] showed that fibroblast proliferation and type I collagen production also increased with increasing platelet concentrations, noting that the best response was at lower plasma pH. These findings explained the association with PRP with improved bone regeneration and soft tissue repair. Since most people have a baseline platelet count of about 200–250 × 10^9^/L, a PRP platelet count of about 1 million/L has become the “benchmark for therapeutic PRP”.

In our protocol, the average number of platelet cells in PRP was 882.5 ± 143.62 × 10^9^/L, which is slightly below the “standard”. However, we found a good therapeutic effect, confirmed by clinical and immunohistochemical data after PRP application. It should be noted that the above concentration (882.5 × 10^9^/L) of platelets was taken as the value determined in 2 mL of the supernatant of cells after the second centrifugation. It is known that the separation of cellular elements by centrifugation allows one to obtain both platelet-poor plasma (PPP) in the upper layer of the supernatant plasma, and rich-as it approaches the border with the erythrocyte sediment. Thus, the maximum platelet concentration is determined in the lowest 1 mL of plasma at the border with the red erythrocyte layer. Since we used 2 mL of plasma, PRP was “diluted”. If only 1 lower ml of plasma is used as PRP, then according to our protocol the platelet count will be about 1660 × 10^9^/L and, possibly, will have a great therapeutic potential. More research is needed to answer this and other PRP questions

We did not use questionnaires, hair-pulling tests, or other subjective instruments to assess the effectiveness of therapy; we used trichoscopy and phototrichogram as non-invasive methods for quantitative analysis of hair growth in patients. In a comparative analysis of phototrichological and morphological data as non-invasive and invasive methods for assessing hair growth before and after treatment, it was confirmed that these methods show unidirectional dynamics, the phototrichogram reliably reflects the morphofunctional state of HF [22].

The positive clinical effect of PRP is associated with growth factors contained in platelets. Growth factors, by binding to receptors sensitive to them, activate signaling pathways that affect the hair growth cycle and HF cell proliferation [23]. Platelet-derived growth factor signals are involved in epidermal–mesenchymal interactions required for hair canal formation and mesenchymal tissue growth [24]. Vascular endothelial growth factor contained in platelets by K. Yano [25] identified as an important mediator of HF growth due to keratinocyte secretion outer root sheaths and dermal papilla fibroblasts, and showed that anagen-associated angiogenesis promotes hair growth and follicle enlargement. It has been reported that PRP induces the proliferation of dermal papilla cells by activating the signaling pathways of fibroblast growth factor 7 (FGF-7) and Wnt/β-catenin [9]. Immunohistochemical studies of the effect of PRP on HF for the qualitative and quantitative assessment of the presence of specific proteins are rare. the level of proliferative activity of HF cells and the number of vessels per mm2 using antibodies to Ki67 and CD31 after application of PRP, an increase in Ki67+ cells and the number of small blood vessels around hair follicles was observed [2,11].

CD34 is primarily considered as a marker of hematopoietic stem and progenitor cells, as well as vascular endothelial progenitor cells and embryonic fibroblasts. In human HF, CD34+ are located below the bulge zone, suprabulbar, as well as in the skin between hair follicles, in the basal cells of the interfollicular epidermis. Throughout the hair cycle, CD34 is expressed during the anagen phase, but not during the catagen and telogen phases. [12]. This is believed to indicate that CD34 function is relevant only to epithelial cells that proliferate, and is also related to the adhesion of root sheath cells to the surrounding stroma. It is worth noting that although human CD34+ cells are not in the bulge zone with other stem cells, they are located in the HF region that exhibits clonogenic activity.

The aim of the study was to assess the effect of PRP on the proliferation of hair follicle cells after treatment, which formed the basis for the selection of different proliferation markers. CD34 could be considered a marker of vascular endothelial cells; however, we were unable to record significant staining of the vascular endothelium in the biopsy material, possibly due to the absence of areas of direct HF vascularization on the sections. In our study, PRP therapy contributed to a significant increase in absolute and relative indicators of CD34+. The marker was present in the interfollicular matrix and in the outer root sheaths of the HF. Kang et al. reported the clinical efficacy of CD34+ cell PRP injections for the treatment of alopecia, showing an increase in hair density and mean hair thickness after 3 and 6 months of follow-up [26]. We have shown an increase in the staining area of CD34+ after PRP therapy, and we assume that the increase in the presence of CD34+ reflects an improvement in the proliferation of HF cells and is beneficial for hair growth and maintenance of the anagen phase.

Studies have shown that the Wnt/β-catenin signaling pathway with the realization of β-catenin accumulation in the cell nucleus with subsequent activation of DNA transcription plays an important role in the development of HF and maintenance of the hair growth cycle. It has been reported that the Wnt10b protein, (the canonical Wnt), promotes hair shaft growth in hair follicle culture, as well as the proliferation of hair matrix cells; Kwack Mi. H. et al. Argue that the initiation of catagen with subsequent transition to telogen occurs in part as a result of specific inhibition of canonical Wnt/β-catenin signaling in hair follicle keratinocytes [8,9].

In vitro, upon treatment with PRP dermal papilla cells, the transcriptional activity of β-catenin increased approximately 2-fold and the level of secreted FGF-7 was higher compared to the control. Activated PRP promotes the formation of hair epithelium and differentiation of hair follicle stem cells through upregulation of β-catenin, strongly expressed in the bulge zone during anagen [10].

In our study, the absolute and relative values of the area of β-catenin expression after treatment significantly increased (*p* = 0.0306 and *p* = 0.0018, respectively). Thus, an increase in the level of β-catenin protein and an increase in its transcriptional activity demonstrate activation of Wnt/β-catenin-signaling pathway and is associated with increased proliferation of hair matrix cells, hair shaft growth, hair epithelium formation, and HF stem cell differentiation during anagen.

In our study, after the application of PRP, the proportion of Ki67+ significantly increased. Ki67 is associated with the phases of the cell cycle, as its expression appears during the G1 phase, increases during the cell cycle, and sharply decreases after mitosis. The Ki67 antigen has a short half-life of no more than 1.5 h and does not accumulate in resting cells. Thus, in immunohistochemical studies, antibodies to the Ki67 antigen reveal proliferating cells at different phases of the cycle and reflect the entire pool of dividing cells. We also found a trend towards an increase in the absolute number of Ki 67+ cells (Δ +125%, *p* = 0.0659).

We did not find the presence of dkk-1 in any of the medicines. Dkk-1 is secreted from dermal papilla cells in response to the presence of dihydrotestosterone. Dkk-1 induces the onset of the catagen phase in the hair growth cycle and regression of the hair follicle by inhibiting the growth of cells of the outer hair sheath and dermal papilla and causing apoptotic cell death [8]. Catagen induction by the dkk-1 molecule occurs in part as a result of blockage in the canonical Wnt/β-catenin signal in HF keratinocytes. The presence of dkk-1 is strongly expressed in follicular keratinocytes at the late anagen stage; then, during the catagen phase, dkk-1 expression decreases and is practically absent in the telogen phase, which confirms the role of dkk-1 in regression of the hair growth cycle. We assume that the absence of dkk-1 expression in our work is due to the short time of the presence of the protein in vivo, and the HFs evaluated in the preparations were in the anagen or telogen phases.

The data on the positive effect of PRP on hair growth in AGA, obtained over the past few years, should prompt clinicians to consider PRP as a valuable alternative to existing treatment methods, given its autologous nature and minimal side effects [27].

Our study has some limitations. Trichoscopic morphological evaluation was objective, but the sample size for immunohistochemistry is small. The average patient follow-up is also short in order to infer the long-term effectiveness of the treatment. We used Rabbit mAb β-catenin to recognize the total level of endogenous protein and do not have quantitative data on nuclear staining and transcriptional activity. Quantifying beta-catenin nuclear staining using an antibody that only binds to nuclear beta-catenin would perhaps be more informative for assessing the effect of PRP on DNA transcription. Nevertheless, our study on skin biopsies confirms the conclusions about the positive effect of PRP on the Wnt/β-catenin signaling pathway obtained earlier in vitro. Thus, further studies are needed with longer follow-up, with more samples. Studies of the dependence of the clinical response on the preparation protocol and the quality of the obtained PRP are also relevant.

## 4. Materials and Methods

Sixty-nine men aged 18 to 53 years (mean age 29.7 ± 1.9 years) were under observation, most of them were men aged 21 to 40 years (81.1%). AGA duration varied from 11 months up to 12 years old and averaged 3.3 ± 0.61 years. 91.3% of patients had II-IV AGA degree according to the Norwood–Hamilton scale.

The inclusion criteria for patients in the study were: male gender; presence of AGA degrees I-IV on the Norwood–Hamilton scale inclusively. As patients who met the inclusion criteria turned up, the method of treatment—PRP, minoxidil, or a combination thereof—was offered to them with a doctor’s explanation of the advantages and disadvantages of each method, the expected clinical effect, and thus the patients were divided into 3 groups.

The criteria for excluding patients from the study were: the presence of AGA of the sixth degree and higher on the Norwood–Hamilton scale; the presence of laboratory-confirmed hyperandrogenism; the presence of inflammatory processes on the scalp; the use of medicines for external use that stimulate hair growth; taking medicines that affect the exchange of steroid hormones (finasteride, dutasteride); the presence of endocrinopathies that affect the exchange of steroid hormones; hair transplant. For patients receiving PRP therapy, additional exclusion criteria were: taking medicines that lower platelet levels (aspirin or other nonsteroidal anti-inflammatory medicines); the presence of leukocytosis or thrombocytopenia in blood tests; the presence of side effects from treatment; a tendency to form keloid or hypertrophic scars. Informed consent was obtained from all patients for examination and treatment the study was approved 13.09.2017, record 114/1 by local ethical committee Saint Petersburg state budgetary healthcare institution “Clinical infectious diseases hospital n.a. S.*p*. Botkin”, an independent ethical committee.

Patients of the first group received PRP injections, patients of the second group received PRP injections in combination with applications of 5% minoxidil solution, patients of the third group-applications of 5% minoxidil solution only. Minoxidil was applied by patients according to the standard method-on dry scalp daily, 2 times a day, in an amount of 1 mL, without subsequent rinsing, for 4 months. The course of PRP injections in patients of the main and the comparison group consisted of 4 procedures with an interval of 1 month. The work was carried out in the design of a prospective, controlled, randomized study. All patients were followed up for 4 months. The pre-treatment observation groups did not differ in age, AGA degree, and morphometric parameters.

Control measurements were taken before the start of treatment and after 4 months. In patients of group 3, control was carried out 4 months after the start of treatment with minoxidil, in patients in groups 1 and 2-1 month after the 4th procedure of PRP therapy. The evaluator was blinded, the analysis of the data was carried out without mentioning which patient the slide belongs to, to which study group and to which period the slide belongs (before or after treatment).

Blood sampling to obtain PRP is carried out by venipuncture into 2 vacuum sterile 9-mL tubes with an anticoagulant (3.8% sodium citrate solution at a ratio of 10:1), a total of 18 mL of venous blood was obtained. Twice centrifugation was carried out. The first for 5 min at 570 g, the second for 10 min at 1200 g. As PRP after the second centrifugation, 2 mL of the lower part of the supernatant from each tube was used; plasma separation was performed manually. An official solution of calcium chloride in a ratio of 1:20 was used as an activator. The scalp surface was treated with chlorhexidine solution; no local anesthesia was applied. The received PRP in the amount of 4 mL was injected into the scalp by microinjection, intradermally, approximately 0.15 mL per injection. The average number of platelets in each ml of PRP was 882.5 ± 143.62 × 10^9^/L, to calculate the number of platelet cells in PRP obtained according to this protocol, a Sysmex XN 2000 hematology analyzer was used.

The clinical efficacy of the therapy was assessed by the dynamics of morphometric indicators of hair growth. Trichological study was performed using a digital video camera “Aramo S” from “AramHuvisCo, Ltd.” (Seongnam-si, Korea) and the TrichoSciencePro v 1.3RUS computer program. The control points were marked with a tattoo mark and were located in the parietal zone subjected to AGA. In the area of control measurements, the hair was shaved 48 h before the study and immediately before the trichoscopy and phototrichogram were stained with dark hair dye RefectoCil from “GW Cosmetics GmbH” (Leopoldsdorf, Austria), which improves contrast and promotes more accurate counting. Hair density was determined per 1 cm^2^, the proportion of vellus and telogen hairs was expressed in %, and the average diameter of all hairs was expressed in microns.

The average hair density in patients before treatment was 381–479/cm^2^, seemed too high for AGA or even normal scalp. According to our preliminary data, the average hair density of the adult population in St. Petersburg and the region (which is the Northwestern part of Europe) is about 540–600/cm^2^, the average hair diameter is 52–60 μm, which may differ from the values these indicators of hair growth in people from other regions, such as the Mediterranean region. Thus, the hair density 381–479/cm^2^ is reduced for our region, as well as the proportion of vellus hair (which in our patients was 34–51% before treatment) and the average diameter (38.8–39.8 μm before treatment). These parameters influence the visual assessment of the degree of hair loss and the severity of the clinical manifestation of AGA.

To assess the effects of the therapy on the morphology of hair after treatment, the quartiles of the distribution of dynamics were calculated for patients of all groups in aggregate for each of the indicators: Hair density, average hair diameter, proportion of vellus, and telogen hair. The results of treatment of a quarter of patients (within the first quartile, Q1) with the lowest dynamics of the hair growth index were considered to have no effect on this indicator. In cases of dynamics within the interquartile range, an improvement in the indicator was considered. The results of treatment of a quarter of patients with the best dynamics of hair growth index (within the third quartile, Q3) were considered a significant improvement in this indicator. 

When evaluating the effectiveness of treatment in cases of significant improvement in two or more indicators of hair growth in conjunction with an improvement in at least one indicator, the therapy was considered highly effective; with an improvement in any three indicators of hair growth or a significant improvement in one indicator in combination with an improvement in any other, a positive treatment result was considered; the rest of the cases were assessed as the absence of a positive clinical effect (with an improvement in only two or less indicators).

Skin biopsies for immunohistochemistry were obtained from 8 patients before and after PRP therapy. Skin biopsies for immunohistochemistry were obtained from 8 patients before and after treatment. Skin biopsy was performed under local anesthesia with 1% lidocaine solution with epinephrine. The material was taken with a biopsy punch with a diameter of 4 mm. Immunohistochemical study was performed on vertical paraffin sections 5 μm thick. To assess the proliferative activity of HF cells, monoclonal antibodies to Ki67 (Cellsignalingtechnology, Danvers, MA, USA; 1: 400), monoclonal antibodies to Beta-catenin (Cellsignalingtechnology, Danvers, MA, USA; 1: 100), monoclonal antibodies to CD34 (Cellsignalingtechnology, Danvers, MA, USA; 1:50), to assess the effect of polyclonal antibodies to DKK-1 on the growth cycle of HF (Bioss Antibodies Inc., Woburn, MA, USA, 1: 250). For the study, the protocol recommended by the manufacturer was used.

Digital microscopy and morphometry included a qualitative and quantitative assessment of the results of immunohistochemical studies on photomicrographs obtained using a microscopic imaging system consisting of an Olympus CX31 microscope, an Olympus BX46 digital camera, and CellSens 47 Entry software. Fields of view with tissue and staining defects, as well as artifacts from photography were excluded. The photographs were taken at a magnification of ×400, ×200 (eyepiece ×10, objective ×40 and ×20). Quantification of immunopositive cells and the area of expression of the studied molecules were carried out in 10 randomized fields of view at a magnification of ×400, not less than 3 sections from each sample. The expression rates of the markers under study were calculated using the VideoTest-Morphology 5.0 software (“VIDEOTEST”, St. Petersburg, Russia).

In each section, the total area of the preparation (μm^2^), the total area of protein expression (μm^2^), and the relative area of expression (calculated as the ratio of the area of immunopositive cells to the total area of the preparation) were assessed. The Ki67 index was calculated as the ratio of the number of immunopositive nuclei to the total number of nuclei in the hair follicle, expressed as a percentage.

Statistical processing and visualization of the results were carried out using the R programming language and the Coin and Ggplot2 libraries. To display the distribution center, the arithmetic mean with indication of the confidence interval was used. To check the relationship between the nominative data, Fisher’s exact test was used (a strict analogue of the χ^2^ test for small samples). To check the relationship between quantitative data, the nonparametric paired Wilcoxon t-test was used. For all statistical tests, the *p*-value was calculated—the probability of error upon rejection of the null hypothesis (the probability of a type I error), *p* < 0.05 was considered statistically significant.

## 5. Conclusions

Improvement of expression of β-catenin, Ki67 and CD34 proteins after PRP therapy characterizes processes that positively affect hair morphology. The effect of PRP on cell proliferation, apoptosis, prolongation of the anagen phase of the HF life cycle is manifested by such morphological effects as a decrease in the proportion of telogen and vellus hair, an increase in the number of hairs, an increase in the average hair diameter. Our results of clinical and immunohistochemical studies are consistent with previously published results and data on the molecular and biological effects of PRP on HF obtained in vitro. Comparative evaluation of treatment results in the case of monoxidil monotherapy and PRP monotherapy did not reveal differences only in terms of hair density. For other indicators, the effectiveness of PRP was significantly higher, *p* ≤ 0.0243, which allows us to consider PRP-therapy as a promising method for treating AGA. The effects of complex therapy significantly exceeded the effects of PRP-therapy and the effects of minoxidil; the complex method of treatment can be successfully used in patients with AGA up to degree 5 on the Norwood–Hamilton scale.

These effects of PRP on HF are possible under the condition of reversibility of pathological processes and are not specific for AGA, which makes further research on the possible effect of PRP on the expression of specific proteins that trigger pathological signaling pathways relevant.

## Figures and Tables

**Table 1:** Dynamics of morphometric indicators of hair growth.

Variable	Unit of Measurement	Monitoring Groups								
		Complex Therapy (PRP + Minoxidil)			PRP			Minoxidil		
		Before Treatment	After Treatment	Dynamics of Change	Before Treatment	After Treatment	Dynamics of Change	Before Treatment	After Treatment	Dynamics of Change
Hair density on 1 cm^2^	Abs.	408.4 ± 43.6	539.6 ± 52.1	131.2	381.5 ± 45.4	426.1 ± 50.1	44.6	479.3 ± 51.5	554.8 ± 53.5	75.5
	∆(%)			32%			12%			16%
	*p*-value			0.00004			0.000067			0.00073
Share of vellus hair, %	Abs.	51.8 ± 6.3	36.5 ± 7.4	−15.3	49.6 ± 7.3	41.0 ± 7.7	−8.6	34.4 ± 5.3	35.2 ± 6	0.8
	∆(%)			−30%			−17%			2%
	*p*-value			0.00082			0.002225			0.7647
Average diameter of all hair, μm	Abs.	38.8 ± 4.2	48.8 ± 5.1	10.0	39.8 ± 3.5	44.4 ± 4.5	4.6	39.3 ± 1.9	40 ± 2.9	0.7
	∆(%)			26%			12%			2%
	*p*-value			0.00004			0.001947			0.338
Share of telogen hair, %	Abs.	50.4 ± 7.3	30.7 ± 7.4	−19.7	42.0 ± 6.4	35.3 ± 7.0	−6.8	42.8 ± 6.7	44.9 ± 6.9	2.1
	∆(%)			−39%			−16%			5%
	*p*-value			0.00008			0.02836			0.338

**Table 2:** Comparison of the effectiveness of treatment in observation groups 1 and 3.

Change in Variable (Abs.)	Monitoring Groups		Comparative Evaluation of Efficacy in Groups 1 and 3
	1 (PRP)	3 (Minoxidil)	
Hair density on 1 cm^2^	44.6	75.5	*p* = 0.1968
Share of vellus hair, %	−8.6	0.8	*p* = 0.0069
Average diameter of all hair, μm	4.6	0.7	*p* = 0.0248
Share of telogen hair, %	6.8	2.1	*p* = 0.0311

**Table 3:** Comparison of the effectiveness of treatment in observation groups 1 and 2.

Change in Variable (Abs.)	Monitoring Groups		Comparative Evaluation of Efficacy in Groups 1 and 2
	1 (PRP)	2 (PRP + Minoxidil)	
Hair density on 1 cm^2^	44.6	131.2	0.0001
Share of vellus hair, %	−8.6	−15.3	0.0715
Average diameter of all hair, μm	4.6	10.0	0.0071
Share of telogen hair, %	−6.8	−19.7	0.0025

**Table 4:** Comparison of the effectiveness of treatment in observation groups 2 and 3.

Change in Variable (Abs.)	Monitoring Groups		Comparative Evaluation of Efficacy in Groups 2 and 3
	2 (PRP+ Minoxidil)	3 (Minoxidil)	
Hair density on 1 cm^2^	131.2	75.5	*p* = 0.0347
Share of vellus hair, %	−15.3	0.8	*p* = 0.0009
Average diameter of all hair, μm	10.0	0.7	*p* = 0.00001
Share of telogen hair, %	−19.7	2.1	*p* = 0.00003

**Table 5:** Dynamics of hair growth indicators after treatment by quartiles of growth distribution.

Variable	Minimum Value (%)	Q_1_ (%)	Median (%)	Q_3_ (%)	Maximum Value (%)
Hair density on 1 cm^2^	−57	5	20	44	127
Average diameter of all hair, μm	−46	−3	13	28	177
Share of vellus hair, %	230	15	−19	−41	−74
Share of telogen hair, %	960	14	−17	−48	−90

**Table 6:** Assessment of clinical efficacy.

Effectiveness	Monitoring Groups						Total
	1 (PRP)		2 (PRP + Minoxidil)		3 (Minoxidil)		
	Abs.	%	Abs.	%	Abs.	%	
Highly efficient	9	36	14	64	0	0	23
Positive clinical effect	7	28	8	36	13	59	28
Lack of clinical effect	9	36	0	0	9	41	18

**Table 7:** Dynamics of immunohistochemical indicators.

Variable	Before	After	Dynamics of Change		*p*-Value
			Abs.	∆ (%)	
CD 34					
Total area	70,883 ± 8420	65,630 ± 7928	−5253	−7%	0.3419
Protein Expression Area	2513 ± 919	9721 ± 2413	7208	287%	0.0001
Relative area,%	3.6 ± 1.20	15.4 ± 4.3	11.8	325%	0.0003
Ki67+					
Qty. of Ki 67+ cells	16.9 ± 6.9	38.0 ± 21.9	21.1	125%	0.0659
Total number of basal cells of HF	248.4 ± 42.2	174.7 ± 28.5	−73.7	−30%	0.0047
% Ki 67+	6.8 ± 2.8	19.8 ± 9.3	13.0	191%	0.0111
β-catenin					
Total area	24,444 ± 6758	28,433 ± 9093	3989	16%	0.4625
Protein Expression Area	2727 ± 1584	7238 ± 3812	4511	165%	0.0306
Relative area,%	11.3 ± 4.2	22.2 ± 5.3	10.9	96%	0.0018
